# Colchicine use after acute myocardial infarction is associated with reduced dialysis dependence in advanced chronic kidney disease

**DOI:** 10.1038/s41598-026-54682-y

**Published:** 2026-06-04

**Authors:** Cheng-Chun Wei, Ming-Hsien Tsai, Yu-Wei Fang, Meng-Ting Chen, Hung-Hsiang Liou, Yen-Chun Huang, Kuan-Yu Chen

**Affiliations:** 1 Division of Cardiology, Department of Internal Medicine, Shin-Kong Wu Ho-Su Memorial Hospital, Taipei, Taiwan; 2 Department of Medicine, Fu Jen Catholic University, New Taipei City, Taiwan; 3 Division of Nephrology, Department of Internal Medicine, Shin-Kong Wu Ho-Su Memorial Hospital, Taipei, Taiwan; 4 Department of Digital Medicine, Shin-Kong Wu Ho-Su Memorial Hospital, Taipei, Taiwan; 5 Department of Healthcare Management, National Taipei University of Nursing and Health Sciences, Taipei, Taiwan; 6 Division of Nephrology, Department of Internal Medicine, Hsin-Jen Hospital, New Taipei City, Taiwan; 7 Department of Artificial Intelligence, Tamkang University, New Taipei City, Taiwan; 8 Division of Cardiology, Department of Internal Medicine, Taipei City Hospital, Zhongxing Branch, 106 Taipei, Taiwan

**Keywords:** Colchicine, Advanced chronic kidney disease, Acute myocardial infarction, Dialysis dependence, Major adverse cardiovascular event, Mortality, Cardiology, Diseases, Medical research, Nephrology

## Abstract

Evidence-based treatments to improve renal outcomes in patients with advanced chronic kidney disease (CKD) after acute myocardial infarction (AMI) are limited. We aimed to evaluate the clinical effects of colchicine therapy after AMI in these patients. This retrospective cohort study used data from the TriNetX US Collaborative Network and included adults with an estimated glomerular filtration rate ≤ 45 mL/min/1.73 m^2^ who were newly diagnosed with AMI between January 1, 2012, and December 31, 2022. Patients were grouped by colchicine use within 1 month after AMI and matched 1:1 by propensity score (n = 1,200 per group). Cox proportional-hazards and Kaplan–Meier analyses assessed outcomes. The primary outcome was dialysis dependence; secondary outcomes included major adverse cardiac events (MACE) and mortality. A landmark analysis was conducted for different colchicine initiation times after AMI. Over a maximum follow-up period of 4 years, colchicine use within one month was associated with reduced risks of dialysis dependence (hazard ratio (HR), 0.65; 95% confidence interval (CI) 0.53–0.79) compared to non-use. However, no significant between-group difference was observed in the risk of MACE (HR 1.04; 95% CI 0.94–1.15) and all-cause mortality (HR 1.03; 95% CI 0.87–1.22). These results were consistent in the landmark analysis. Notably, initiation of colchicine within two weeks was associated with a lower risk of all-cause mortality (HR 0.85; 95% CI 0.73–0.99) in landmark analyses. In patients with advanced CKD experiencing AMI, early colchicine therapy may reduce the risks of dialysis dependence. Any potential survival benefit remains uncertain and should be confirmed in prospective studies.

**Supplementary Information:**

The online version contains supplementary material available at 10.1038/s41598-026-54682-y.

## Introduction

Chronic kidney disease (CKD) is a global public health crisis, affecting more than 800 million individuals and substantially contributing to morbidity, mortality, and health-care costs^1^. Patients with advanced CKD have a high risk of cardiovascular events, particularly acute myocardial infarction (AMI), which is a leading cause of death in this population^2^. Despite therapeutic advances, post-AMI prognosis in patients with advanced CKD remains poor and is characterized by rapid progression to end-stage renal disease and high hospitalization and mortality rates^3,4^. The concept of “cardiorenal syndrome” indicates the complex association between the cardiovascular and renal systems, highlighting the pressing need for innovative strategies aimed at improving outcomes in both domains^5^. AMI is not merely a localized coronary event but a systemic inflammatory insult that triggers a cascade of pathophysiological responses affecting multiple organ systems^6^. In addition to causing recurrent cardiovascular complications, AMI leads to progressive renal dysfunction, particularly in patients with underlying CKD^7^.

Current evidence indicates that inflammation plays a central role in the progression of both cardiovascular disease and CKD^8,9^. Inflammation leads to atherosclerotic plaque destabilization, post-AMI ventricular remodeling^10^, and renal fibrosis, which results in functional decline^11^. Among the various inflammatory pathways implicated, activation of the NOD-like receptor family pyrin domain containing 3 inflammasome is a key mechanism linking innate immune responses to organ damage^12^. Although treatments targeting traditional risk factors such as hypertension, dyslipidemia, and diabetes may be beneficial for patients with early-stage CKD, these approaches are less effective in patients with advanced disease^13^. Few therapies directly target chronic inflammation, a characteristic feature of advanced CKD, prompting increasing interest in anti-inflammatory agents to decelerate progressive organ dysfunction.

Colchicine, a well-established and cost-effective anti-inflammatory agent, effectively reduced recurrent cardiovascular events in large-scale randomized trials involving patients with coronary artery disease^14,15^. For example, the Colchicine Cardiovascular Outcomes Trial (COLCOT)^14^ and the Low-Dose Colchicine 2 (LoDoCo2) trial^15^ have reported that low-dose colchicine (0.5–0.6 mg daily) reduces the incidence of major adverse cardiovascular events (MACEs) in patients with AMI and those with chronic coronary disease, respectively. However, these studies either excluded patients with advanced CKD or included a small number of these patients; therefore, the effects of colchicine on renal outcomes in this high-risk population remain unclear^16^. Moreover, very few studies have investigated whether colchicine, through its anti-inflammatory properties, can decelerate renal disease progression or reduce dialysis dependence after MACEs.

In this study, we investigated the effects of post-AMI colchicine therapy on clinical outcomes in patients with advanced CKD. By analyzing data from TriNetX and conducting propensity score matching (PSM), we provide actionable insights for the management of patients with advanced CKD after cardiac events.

## Methods

### Data source

The TriNetX US Collaborative Network is a health-care research platform that connects hospitals, pharmaceutical companies, and researchers to accelerate medical research and improve patient care. It features a large, continually updated database that offers anonymized patient data derived from medical records. The platform enables efficient cohort identification, feasibility assessment, and advanced statistical analyses for robust comparisons. TriNetX generates real-world evidence by analyzing structured electronic health record (EHR) data (demographics, diagnoses, procedures, prescriptions, labs, and vital signs) from over 220 healthcare organizations in 30+ countries, mainly academic centers and community hospitals. Claims data and links to external registries are rare and not standardized across the network. TriNetX enables investigators to identify and analyze patient groups for clinical trials, streamlining recruitment. Its advanced visualization and analytical tools help users explore trends, treatments, and outcomes. All data are de-identified, with only aggregated counts and summary statistics available, ensuring patient privacy. Because TriNetX supplies only aggregated, de-identified data, its use was exempt from informed consent by the Western Institutional Review Board. This dataset has previously been utilized for research purposes and has also been published in various scientific literature. It has contributed to multiple studies and appears in several academic publications^17–19^.

### Ethics approval and consent to participate

This study followed the International Conference on Harmonization Good Clinical Practice guidelines, the Declaration of Helsinki, and all relevant regulations for noninterventional and observational studies. The study protocol was approved by the Ethics Review Board of Shin Kong Wu Ho-Su Memorial Hospital, Taiwan (approval number: 20240812R). The requirement for informed consent was waived because TriNetX offers deidentified data.

### Study design and cohort definitions

**Fig. 1:**
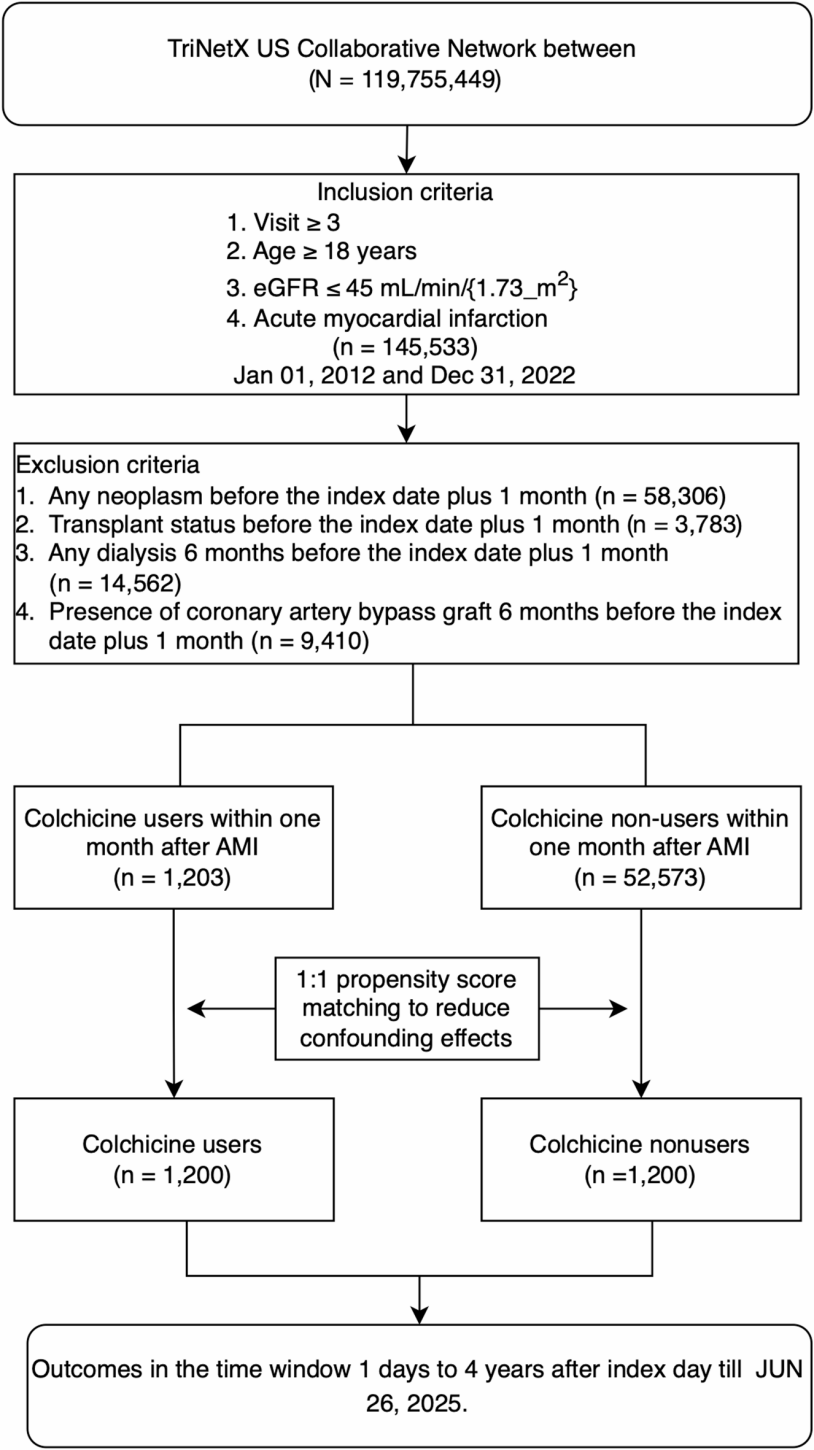
Flowchart depicting patient selection. eGFR, estimated glomerular filtration rate.

This was a retrospective cohort study. We investigated the outcomes of post-AMI colchicine therapy in patients with advanced CKD. Relevant data were obtained from TriNetX, which includes the medical records for over 119 million individuals. Figure 1 presents a flowchart depicting patient selection. The inclusion criteria were as follows: having ≥ 3 clinical visits; being aged ≥ 18 years; having an estimated glomerular filtration rate (eGFR) of ≤ 45 mL/min/1.73 m^2^ (CKD stages 3b to 5); and consequently, having an AMI event between January 1, 2012, and December 31, 2022. The index date was defined as the date of the AMI attack. AMI was defined using International Classification of Diseases, 10th Revision (ICD-10) codes I21.x, corresponding to clinically diagnosed ST-segment elevation myocardial infarction and non–ST-segment elevation myocardial infarction. Conditions such as pericarditis or other non-ischemic causes of troponin elevation were not included in cohort identification. Patients were excluded if they had a diagnosed malignancy or underwent organ transplantation before the index date plus one month. We also excluded patients receiving any dialysis procedures (hemodialysis and peritoneal dialysis) or those who underwent coronary artery bypass grafting within six months before the index date plus one month to reduce heterogeneity related to surgical complexity, perioperative acute kidney injury risk, and distinct postoperative mortality trajectories.

After applying the eligibility criteria, patients were classified according to colchicine exposure within one month after the index AMI. Colchicine users were defined as those with at least one prescription record during this post-AMI window, whereas patients without a colchicine prescription during the same period were classified as nonusers. In total, 1,203 colchicine users and 52,573 nonusers were identified prior to propensity score matching (PSM). The study design is illustrated in Fig. S1.

To minimize confounding, 1:1 PSM was performed, resulting in two well-balanced groups. Study outcomes were tracked for periods ranging from 1 day to 4 years after the index date, with follow-up continuing until June 26, 2025. This rigorous patient selection and matching process supports a robust comparative effectiveness assessment by closely mimicking a randomized controlled trial within an observational dataset, thereby reducing biases from baseline between-group differences. Cohort definitions are detailed in Method S1.

### Covariates

Baseline clinicodemographic data were systematically collected for the 1 years preceding the index date. Demographic characteristics included age on the index date, sex, race (White, African American, Asian, and others), and body mass index (BMI). Clinical characteristics included dyslipidemia, diabetes mellitus, hypertensive diseases, atrial fibrillation and flutter, chronic lower respiratory diseases, nicotine dependence, cardiomyopathy, other cardiac arrhythmias, heart failure, tobacco use, alcohol-related disorders, cerebrovascular diseases, pulmonary heart disease, chronic rheumatic heart diseases, thyroid disorders, liver cirrhosis, and fatty liver disease. We further recorded detailed information on the use of various medications, such as insulin, noninsulin glucose-lowering drugs, renin–angiotensin system blockers, statins, fibrates, antithrombotic agents, diuretics, beta-blockers, calcium channel blockers, and peripheral vasodilators. Laboratory parameters included eGFR, sodium, potassium, calcium, hemoglobin, alanine transaminase, aspartate transaminase, albumin, phosphate, low-density lipoprotein cholesterol, high-density lipoprotein cholesterol, triglycerides, hemoglobin A1c, troponin I, and urinary protein-to-creatinine ratio (UPCR). The values of laboratory data were selected from the measurements taken closest to the index date, ensuring they most closely corresponded to that index date in time. Incorporating all baseline characteristics into the primary PSM ensured data comprehensiveness, supporting study validity and comparability between colchicine users and nonusers throughout follow-up. Code definitions of baseline characteristics are detailed in Method S1.

### Study outcomes

The primary outcome was the development of dialysis dependence, whereas the secondary outcomes were post-index date MACE components (myocardial infarction, stroke, heart failure, and death) and all-cause mortality. In addition, the following outcomes were assessed: stroke, recurrent AMI, atrial fibrillation or flutter, heart failure, and cardiac arrest. These outcomes were selected to determine the medium- to long-term risks and clinical events experienced by patients with advanced CKD after AMI. Outcomes were monitored until the first occurrence of the specified events, death, loss to follow-up, or study end date (June 26, 2025), whichever occurred first. The ICD-10 codes for outcomes are detailed in Method S1.

### Sensitivity analysis

To evaluate the robustness of our findings, five sensitivity analyses were performed. First, we used stepwise PSM models, adjusted for multiple confounders to minimize bias and isolate the individual effects of the variables under study. Model 1 matched on age, sex, race, and BMI; model 2 added baseline comorbidities; and model 3 further included baseline medication use. Second, we evaluated risk profiles at different time points to address potential violations of the proportionality assumption, determining whether the results were consistent over time. Third, we performed a landmark analysis to assess the outcomes of colchicine use at 1, 2, and 3 weeks after AMI, providing additional insights into the timing and durability of its therapeutic effects. Fourth, we analyzed outcomes after excluding patients who used colchicine in the three months before the index date, limiting the analysis to those without recent pre-index colchicine exposure. Finally, we incorporated a broader and more detailed set of gout- and inflammation-related baseline data into the propensity score matching process, including gout characteristics, nonsteroidal anti-inflammatory drug use, and baseline serum urate, C-reactive protein, and erythrocyte sedimentation rate levels to better reflect patients’ pre-index clinical status. These analyses strengthened the validity of our conclusions and offered a comprehensive understanding of the data.

### Statistical analysis

Categorical data are presented as count (%) values, whereas continuous variables are presented as mean ± standard deviation. Between-group differences were determined using the standardized mean difference (SMD) value, with values of < 0.2, 0.2–0.5, and 0.51–0.8 indicating small, moderate, and large effects, respectively^20^. PSM was performed to establish comparable groups, minimizing baseline confounding and enabling accurate comparisons. Baseline demographic, clinical, and laboratory characteristics were evaluated, with an SMD value of < 0.1 indicating well-balanced groups^21^. During PSM, age on the index date was treated as a continuous variable, whereas sex, race, lifestyle factors, comorbidities, and medication use were treated as categorical variables (present/absent). Laboratory parameters were marked as present if available or as absent if missing within the specified time window. Propensity scores were generated using logistic regression and matched using greedy nearest-neighbor matching with a 0.1 caliper to ensure group balance for treatment effect analysis (Matching details are provided in Method S2).

Cox proportional-hazards regression analysis was performed to calculate hazard ratios (HRs) and corresponding 95% confidence interval (CIs) for comparing clinical outcomes between colchicine users and nonusers. The primary analysis utilized unadjusted models within the matched cohorts. The proportionality assumption was tested using the generalized Schoenfeld method in the TriNetX platform. Kaplan–Meier curves were generated to estimate event-free probabilities. Subgroup analyses were performed to analyze treatment effects by sex (male or female); race (White, African American, Asian, or others); eGFR (29.9–45, 15–30, and < 15 mL/min/1.73m^2^); BMI (≥ 30 or < 30 kg/m^2^); glycated hemoglobin percentage (≥ 7% or < 7%); and comorbidities (present or absent), such as heart failure, dyslipidemia, diabetes mellitus, hypertensive disease, pulmonary heart disease and gout. All subgroups in the study were subjected to PSM using all baseline variables to create well-balanced and comparable pairs. Because the TriNetX platform has limited support for interaction p-values, the p-value for heterogeneity was calculated instead to assess whether the effect of colchicine on dialysis dependence differed significantly between subgroups. Heterogeneity among subgroups was evaluated using Cochran’s Q test and I^2^ statistics^22^. A random-effects model was applied when substantial heterogeneity was detected.

All statistical analyses were performed using the TriNetX platform. No multiple imputation was performed; analyses were based on available data. Statistical significance was defined as a two-sided *P* value of < 0.05. All figures were generated using R (version 4.4.2; R Foundation for Statistical Computing, Vienna, Austria).

## Results

### Baseline characteristics

Initially, Table 1 comprised a large cohort of 1,203 colchicine users and 52,573 nonusers, which exhibited significant baseline imbalances across multiple demographic and clinical characteristics. After PSM, SMDs were < 0.1 for all variables, indicating good balance between the matched groups (1,200 patients in each group), except for BMI modeled as a continuous variable, which remained slightly imbalanced (SMD = 0.104); however, BMI in categorical form achieved acceptable balance (all SMDs < 0.1), supporting minimized selection bias overall. The mean age was approximately 65 years, with a majority of male participants (62–64%). Patients exhibited high prevalence of comorbidities, including hypertensive diseases (86%), heart failure (60–61%), and diabetes mellitus (52%). Significant cardiovascular risk factors were evident, with dyslipidemia affecting around 60% of participants and substantial rates of atrial fibrillation (32–34%). Laboratory parameters revealed advanced chronic kidney disease, with a mean eGFR of 34–36 mL/min/1.73m^2^, indicating advanced renal impairment.

**Table 1:** Baseline characteristics of study population before and after propensity score matching^&^

Parameteres	Before matching			After matching		
	Colchicine (n = 1,203)	Non-Colchicine (n = 52,573)	SMD	Colchicine (n = 1,200)	Non-Colchicine (n = 1,200)	SMD
Age at Index (years) , mean ± SD	65.2 ± 13.6	65.8 ± 13	0.042	65.2 ± 13.6	65.3 ± 12.9	0.006
Male, *n* (%)	748 (62.2)	26,771 (50.9)	0.229	745 (62.1)	769 (64.1)	0.041
BMI (kg/m^2^), mean ± SD	31.9 ± 8.6	30 ± 7.9	0.234	31.9 ± 8.6	31 ± 7.9	0.104
> 35, *n* (%)	832 (24.9)	9,943 (18.9)	0.146	299 (24.9)	176 (23)	0.045
20–35, *n* (%)	602 (50)	27,462 (52.20	0.044	600 (50)	616 (51.3)	0.027
< 20, *n* (%)	43 (3.6)	3,143 (6.0)	0.113	43 (3.6)	47 (3.9)	0.018
Race, *n* (%)						
White people	606 (50.4)	30,746 (58.5)	0.163	604 (50.3)	607 (50.6)	0.005
African American	374 (31.1)	12,867 (24.5)	0.148	373 (31.1)	348 (29)	0.045
Asian	85 (7.1)	2,708 (5.2)	0.080	85 (7.1)	97 (8.1)	0.038
Other race	20 (1.7)	1,308 (2.5)	0.058	20 (1.7)	27 (2.3)	0.042
Lifestyle, *n* (%)						
Tobacco use	62 (5.2)	2,408 (4.6)	0.027	62 (5.2)	80 (6.7)	0.064
Alcohol related disorders	59 (4.9)	2,364 (4.5)	0.019	58 (4.8)	60 (5)	0.008
Nicotine dependence	205 (17)	9,795 (18.6)	0.042	205 (17.1)	205 (17.1)	< 0.001
Comorbidity , *n* (%)						
Dyslipidemia	733 (60.9)	28,648 (54.5)	0.131	730 (60.8)	735 (61.3)	0.009
Diabetes mellitus	634 (52.7)	27,426 (52.2)	0.011	633 (52.8)	619 (51.6)	0.023
Hypertensive diseases	1,040 (86.5)	42,834 (81.5)	0.136	1,037 (86.4)	1,048 (87.3)	0.027
Cardiomyopathy	215 (17.9)	6,480 (12.3)	0.155	212 (17.7)	223 (18.6)	0.024
Atrial fibrillation and flutter	386 (32.1)	13,148 (25)	0.157	384 (32)	414 (34.5)	0.053
Other cardiac arrhythmias	226 (18.8)	8,414 (16)	0.073	225 (18.8)	237 (19.8)	0.025
Heart failure	724 (60.2)	25,957 (49.4)	0.218	721 (60.1)	733 (61.1)	0.020
Cerebrovascular diseases	189 (15.7)	9,821 (18.7)	0.079	189 (15.8)	195 (16.3)	0.014
Pulmonary heart disease	239 (19.9)	7,435 (14.1)	0.153	237 (19.8)	224 (18.7)	0.028
Chronic rheumatic heart diseases	140 (11.6)	4,722 (9)	0.087	140 (11.7)	154 (12.8)	0.036
Chronic lower respiratory diseases	328 (27.3)	13,082 (24.9)	0.054	327 (27.3)	315 (26.3)	0.023
Disorders of thyroid gland	193 (16)	8,452 (16.1)	0.001	193 (16.1)	186 (15.5)	0.016
Liver cirrhosis	27 (2.2)	1,364 (2.6)	0.023	27 (2.3)	27 (2.3)	< 0.001
Fatty liver	26 (2.2)	848 (1.6)	0.040	26 (2.2)	25 (2.1)	0.006
Medication, *n* (%)						
Insulin	514 (42.7)	21,761 (41.4)	0.027	513 (42.8)	522 (43.5)	0.015
Glucose-lowering drugs (excluding insulin)	189 (15.7)	6,857 (13)	0.076	188 (15.7)	164 (13.7)	0.057
RASB	466 (38.7)	17,068 (32.5)	0.131	463 (38.6)	440 (36.7)	0.040
Statin	734 (61)	27,490 (52.3)	0.177	732 (61)	714 (59.5)	0.031
Fibrates	28 (2.3)	1,175 (2.2)	0.006	28 (2.3)	28 (2.3)	< 0.001
Antithrombotic agents	1,038 (86.3)	40,758 (77.5)	0.229	1,035 (86.3)	1,010 (84.2)	0.059
Diuretics	690 (57.4)	24,042 (45.7)	0.234	687 (57.3)	672 (56)	0.025
Beta-blocker	789 (65.6)	29,493 (56.1)	0.195	786 (65.5)	775 (64.6)	0.019
Calcium channel blockers	469 (39)	18,043 (34.3)	0.097	468 (39)	493 (41.1)	0.043
Peripheral vasodilator	17 (1.4)	866 (1.6)	0.019	17 (1.4)	20 (1.7)	0.020
Laboratory, mean ± SD						
eGFR (mL/min/1.73m^2^)	36.6 ± 27	35.5 ± 27.6	0.039	36.6 ± 27	34.2 ± 25.2	0.090
Sodium (mmol/L)	137.1 ± 4.6	137.3 ± 4.9	0.037	137.1 ± 4.6	137.3 ± 4.8	0.046
Potassium (mmol/L)	4.2 ± 0.7	4.2 ± 0.7	0.013	4.2 ± 0.7	4.2 ± 0.7	0.018
Calcium (mg/dL)	8.9 ± 0.8	8.8 ± 0.9	0.082	8.9 ± 0.8	8.8 ± 0.8	0.048
Hemoglobin (g/dL)	11.6 ± 2.5	11.4 ± 2.6	0.052	11.6 ± 2.5	11.6 ± 2.5	0.032
ALT (U/L)	34.9 ± 61	67.2 ± 322.1	0.140	34.9 ± 61.1	50.5 ± 214.1	0.099
AST(U/L)	47.5 ± 80.9	96.1 ± 522.5	0.130	47.6 ± 81	67.1 ± 274.2	0.097
Albumin (g/dL)	3.5 ± 0.6	3.4 ± 0.7	0.104	3.5 ± 0.6	3.5 ± 0.6	0.036
Phosphate (mg/dL)	4 ± 1.3	4.2 ± 1.7	0.159	4 ± 1.3	4.1 ± 1.4	0.066
LDL (mg/dL)	87.2 ± 40.1	88.8 ± 43.7	0.037	87.1 ± 40.1	84.4 ± 38.1	0.069
HDL (mg/dL)	39.9 ± 17.4	41.1 ± 17.4	0.070	40 ± 17.4	39.4 ± 16.4	0.035
Triglyceride (mg/dL)	145.5 ± 117.9	146.4 ± 121.4	0.008	145 ± 117.3	151.9 ± 109	0.061
Hemoglobin A1c (%)	6.8 ± 2.1	7 ± 2.1	0.105	6.8 ± 2.1	6.8 ± 1.8	0.017
Troponin I (ng/ml)	4.5 ± 20.9	3.5 ± 19.2	0.051	4.5 ± 21	3.7 ± 20	0.039
UPCR (mg/g)	1264 ± 2506.9	1143 ± 2511.4	0.048	1264 ± 2506.9	1508.4 ± 3117.4	0.086

^&^The primary propensity score matching includes all parameters in this table to achieve balanced cohorts.

*SD* Standard deviation; *SMD* Standardized mean difference; *BMI* Body mass index; *RASB* Renin-angiotensin system blockader; *eGFR* Estimated glomerular filtration rate; *ALT* Aspartate aminotransferase; *AST* Aspartate aminotransferase; *LDL* Low density lipoprotein; *HDL* High density lipoprotein; *UPCR* Protein/creatinine in urine.

### Association of renal outcomes with colchicine use after AMI in advanced CKD

**Fig. 2:**
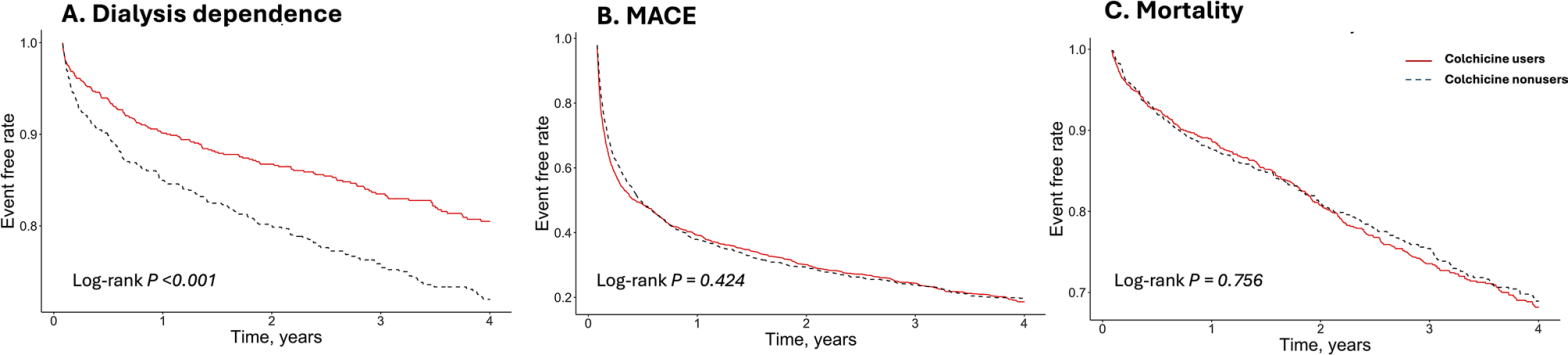
Kaplan–Meier cumulative event-free plots. Rates of (**A**) dialysis dependence, (**B**) major adverse cardiovascular events (MACEs), and (**C**) all-cause mortality in colchicine users and nonusers.

Figure S2 depicts the follow-up time distribution, with colchicine users having a mean follow-up of 874 ± 570 days, compared to 749 ± 858 days for nonusers. Kaplan–Meier curves (Fig. 2A) revealed that post-AMI colchicine use in patients with advanced CKD was associated with a lower risk of dialysis dependence over the 4-year follow-up period (*P* < 0.001), with the difference between users and nonusers becoming increasingly evident over time. This association is reflected in the 4-year follow-up data presented in Table 2, highlighting a 35% lower risk of dialysis dependence in colchicine users had a (13.4% versus 17.4%; HR 0.65; 95% CI 0.53–0.79) than in nonusers.

**Table 2:** Clinical outcomes in advanced chronic kidney disease patients after acute myocardial infraction.

Clinical outcomes	After propensity score matching^&^; with a maximum follow-up of 4-year					
	Colchicine users within one month (n = 1,200)		Colchicine nonusers within one month (n = 1,200)		Colchicine users vs. Nonusers	
	Events	%	Events	%	HR (95%CI)	*P value*
Primary outcome						
Dialysis dependence	161	13.4	209	17.4	0.65 (0.53–0.79)	< 0.001
Secondary outcome						
MACE^#^	817	68.1	726	60.5	1.04 ( 0.94–1.15 )	0.424
Mortality	281	23.4	237	19.8	1.03 ( 0.87–1.22 )	0.756
Specified analysis						
Stroke	115	9.6	133	11.1	0.74 ( 0.58–0.95 )	0.019
AMI	399	33.3	350	29.2	1.03 ( 0.89–1.19 )	0.714
Af and AF	386	32.2	342	28.5	1.02 ( 0.88–1.18 )	0.764
Heart failure	643	53.6	584	48.7	1.01 ( 0.9–1.12 )	0.931
Cardiac arrests	73	6.1	69	5.8	0.92 ( 0.66–1.28 )	0.612^†^

^&^The propensity score matching includes all parameters in Table 1 to achieve balanced cohorts.

^†^This indicate the proportional hazard assumption is violated.

^#^MACE included myocardial infarction, stroke, heart failure, and death.

*HR* Hazard ratio; *CI* Confidence interval; *MACE* Major adverse cardiovascular events; *AMI* Acute myocardial infarction; *Af* Atrial fibrillation; *AF* Atrial flutter.

### Association of cardiovascular outcomes with colchicine use after AMI in advanced CKD

Kaplan–Meier curves (Fig. 2B,C) further indicated that post-AMI colchicine use in patients with advanced CKD did not significantly affect the risk of MACEs (*P* = 0.424) or all-cause mortality (*P* = 0.756) over the 4-year follow-up period. The lack of cardiovascular benefit is supported by the data in Table 2, indicating no significant effect of colchicine use on the risk of MACEs (HR 1.04; 95% CI 0.94–1.15), all-cause mortality (HR 1.03; 95% CI 0.87–1.22), recurrent AMI (HR 1.03; 95% CI 0.89–1.19), atrial fibrillation or flutter (HR 1.02; 95% CI 0.88–1.18), heart failure (HR 1.01; 95% CI 0.90–1.12), or cardiac arrest (HR 0.92; 95% CI 0.66–1.28). However, colchicine significantly reduced the risk of stroke (9.6% vs. 11.1%; HR 0.74; 95% CI 0.58–0.95).

### Results of subgroup analysis

**Fig. 3:**
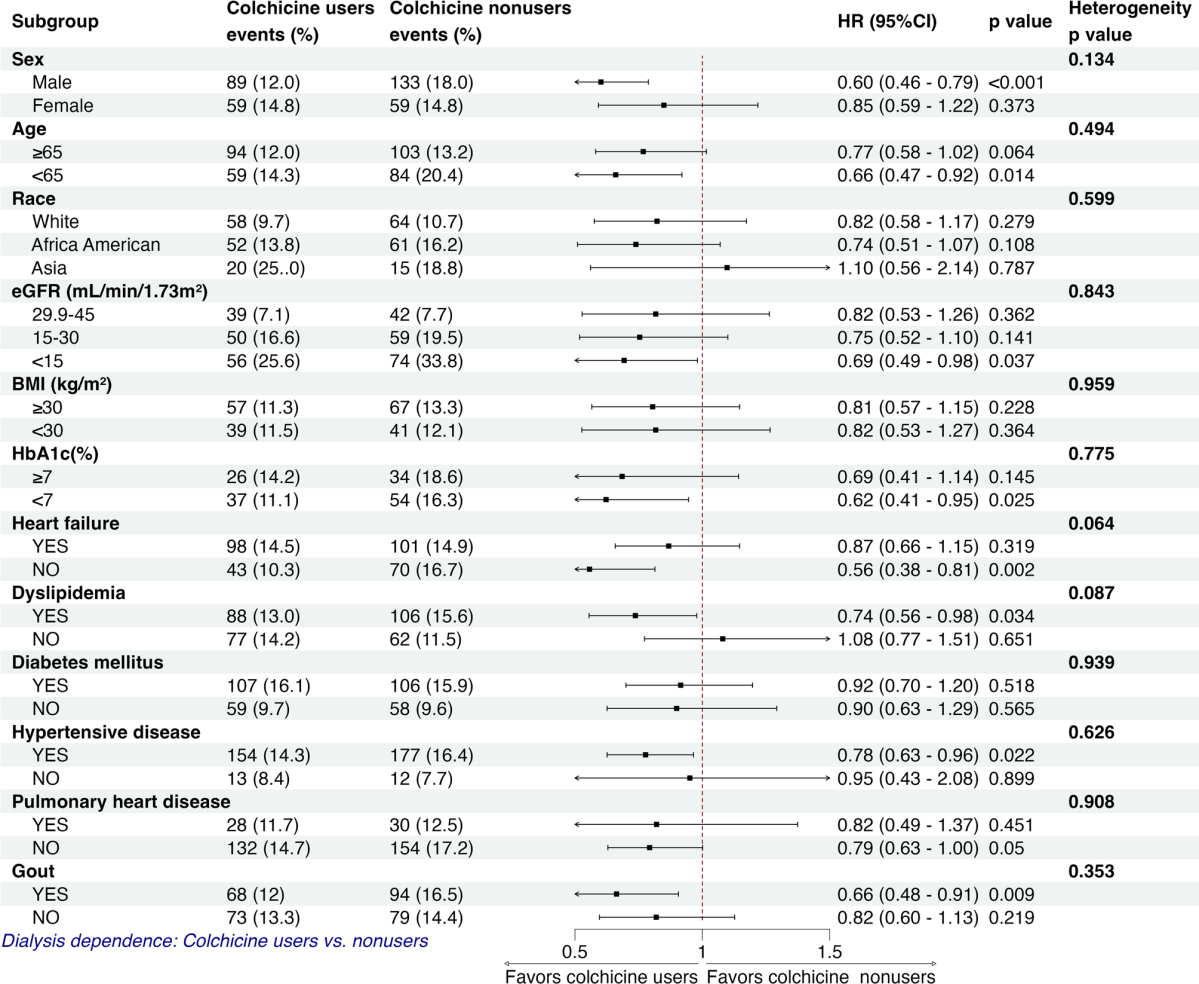
Results of a subgroup analysis. Analysis of dialysis dependence in colchicine users versus nonusers. The vertical line indicates an HR of 1.00. If the lower limit of 95% CI exceeds 1.00, it indicates a notably elevated risk of dialysis dependence. *HR* Hazard ratio; *CI* Confidence interval; *BMI* Body mass index; *HbA1c* Glycated hemoglobin.

Subgroup analyses (Fig. 3) demonstrated that colchicine use was associated with a significantly lower risk of dialysis dependence in several predefined subgroups, including male patients (HR 0.60, 95% CI 0.46–0.79), individuals aged < 65 years (HR 0.66, 95% CI 0.47–0.92), patients with HbA1c < 7% (HR 0.62, 95% CI 0.41–0.95), those without heart failure (HR 0.56, 95% CI 0.38–0.81), patients with dyslipidemia (HR 0.74, 95% CI 0.56–0.98), those with hypertension (HR 0.78, 95% CI 0.63–0.96), and patients with gout (HR 0.66, 95% CI 0.48–0.91). In the eGFR subgroup analysis, colchicine was associated with lower HRs across all categories. This benefit was significant only in patients with eGFR < 15 mL/min/1.73 m^2^ (HR 0.69, 95% CI 0.49–0.98). In the remaining subgroups, the hazard ratios consistently favored colchicine, although statistical significance was not reached. Importantly, there was no significant heterogeneity across subgroups (all P for interaction > 0.05), indicating a consistent renal protective effect of colchicine across the examined populations.

### Landmark analysis

Further landmark analysis in Table 3 shows results consistent with our findings. It demonstrates that initiating colchicine therapy within 2 weeks (HR 0.69; 95% CI 0.55–0.86) or within 3 weeks (HR 0.71; 95% CI 0.57–0.88) after an AMI, but not within the first week, was associated with a significant reduction in the risk of developing dialysis dependence compared to patients who did not receive colchicine. In addition, colchicine use was associated with a lower risk of all-cause mortality when administered within 1 week (HR 0.82; 95% CI 0.68–0.99) and within 2 weeks (HR 0.85; 95% CI 0.73–0.99) following AMI.

**Table 3:** Landmark analysis of clinical outcomes in advanced CKD patients post-AMI.

Clinical Outcomes	Propensity score matching with a follow-up of 4-year					
	Colchicine-treated vs. untreated within 1 week (n = 804 pairs)		Colchicine-treated vs. untreated within 2 weeks (n = 1,099 pairs)		Colchicine-treated vs. untreated within 3 weeks (n = 1,003 pairs)	
	HR (95%CI)	*P value*	HR (95%CI)	*P value*	HR (95%CI)	*P value*
Primary outcome						
Dialysis dependence	0.89 (0.69–1.13)	0.327	0.69 (0.55–0.86)	0.001	0.71 (0.57–0.88)	0.002
Secondary outcomes						
MACE^#^	0.96 ( 0.85–1.07 )	0.431	1.04 ( 0.94–1.14 )	0.456	1.02 ( 0.92–1.13 )	0.695
Mortality	0.82 ( 0.68–0.99 )	0.049	0.85 ( 0.73–0.99 )	0.049	0.99 ( 0.83–1.17 )	0.879
Specified analysis						
Stroke	0.74 ( 0.56–0.99 )	0.044	0.85 ( 0.67–1.1 )	0.212	0.8 ( 0.62–1.04 )	0.095
AMI	0.97 ( 0.83–1.13 )	0.688	1.11 ( 0.97–1.27 )	0.125	1.09 ( 0.94–1.25 )	0.248
Af and AF	1.02 ( 0.85–1.22 )	0.858	1.12 ( 0.96–1.29 )	0.147	1 ( 0.85–1.16 )	0.954
Heart failure	0.97 ( 0.85–1.1 )	0.620	1.05 ( 0.94–1.17 )	0.387	1.02 ( 0.91–1.14 )	0.759
Cardiac arrest	0.88 ( 0.59–1.29 )	0.500	0.78 ( 0.57–1.07 )	0.124	0.94 ( 0.68–1.32 )	0.730

^&^The propensity score matching includes all parameters in Table 1 to achieve balanced cohorts.

^†^This indicate the proportional hazard assumption is violated.

^#^MACE included acute myocardial infarction, stroke, heart failure and death.

*CKD* Chronic kidney disease; *HR* Hazard ratio; *CI* Confidence interval; *MACE* Major adverse cardiovascular events; *AMI* Acute myocardial infarction; *Af/AF* Atrial fibrillation and atrial flutter.

### Adverse events analysis

After PSM, the rates of adverse events were similar between colchicine users and nonusers (Table S1). Myopathy occurred in 2.3% of all users and 1.9% of all nonusers. Bone marrow suppression occurred in 11.5% of the users and 9.2% of the nonusers. Hepatotoxicity occurred in 1.9% of the users and 1.6% of the nonusers. Diarrhea occurred in 11.9% of the users and 10.6% of the nonusers. None of the aforementioned adverse events differed significantly between the two groups.

### Results of sensitivity analysis

Our findings were consistent across different PSM model analyses (Table S2) and time frame analyses (Table S3). However, in Model 1, colchicine use was linked to a higher risk of MACEs, but this association became neutral after adjusting for comorbidities and medications (Model 2 and 3). Moreover, sensitivity analyses that excluded patients with prior colchicine use (Table S4) and, separately, incorporated additional gout- and inflammation-related covariates into the matching process (Table S5) produced results that were consistent with the main analysis. Across these approaches, colchicine use remained associated with a lower risk of dialysis dependence, whereas MACE and all-cause mortality showed largely neutral effects over the four-year follow-up period.

## Discussion

The present study addressed a major knowledge gap regarding the post-AMI management of patients with advanced CKD. The analysis of EHR data from TriNetX and robust PSM revealed that initiating colchicine therapy within 1 month after AMI was associated with a 35% lower hazard of dialysis dependence. In addition, colchicine use was associated with a 26% lower hazard of stroke, which was evaluated as a secondary endpoint. Moreover, a lower all-cause mortality risk was observed in the group receiving colchicine within two weeks after AMI. Although the observational design precludes definitive causal inference, these findings suggest that early colchicine use after AMI may be associated with improved renal outcomes in patients with advanced CKD. Further prospective studies are warranted to validate these findings and to better define the role of colchicine in this high-risk population.

Our study advances current knowledge on the therapeutic potential of colchicine. Large randomized trials, such as COLCOT^14^ and LoDoCo2^15^, have demonstrated the efficacy of colchicine in preventing secondary cardiovascular events in patients with AMI and those with chronic coronary syndrome, respectively. In the COLCOT trial, 4,745 patients who had had myocardial infarction within the previous 30 days were randomized to receive 0.5 mg of colchicine daily or placebo. Over a median follow-up period of 22.6 months, colchicine therapy reduced the composite outcome of cardiovascular mortality, cardiac arrest with successful resuscitation, myocardial infarction, stroke, or urgent revascularization by 23%^14^. Similar cardiovascular benefits were observed in the LoDoCo2 trial, which enrolled more than 5,000 patients with chronic coronary disease^15^. However, these studies largely excluded patients with advanced CKD. A meta-analysis reported potential cardiovascular benefits of colchicine in patients with milder CKD, but it did not specifically evaluate renal outcomes or include a substantial number of patients with advanced disease^3^.

The renoprotective effect of colchicine observed in the present study is consistent with experimental evidence indicating that colchicine inhibits the NOD-like receptor family pyrin domain containing 3 inflammasome^23^, a key mediator of both cardiovascular disease and kidney injury^24,25^. This mechanism is particularly relevant in the post-AMI setting, where systemic inflammation can exacerbate kidney dysfunction through multiple pathways, such as by increasing oxidative stress, causing endothelial dysfunction, and inducing profibrotic signaling^26^. Our finding of a reduced stroke risk with colchicine, despite its neutral effects on other cardiovascular outcomes, is consistent with that of a meta-analysis reporting that colchicine may exert a more pronounced effect on cerebrovascular events than on other cardiovascular outcomes^27^. Together, the findings implicate inflammation in the pathogenesis of stroke in patients with advanced CKD, which is characterized by an increased burden of cerebrovascular disease^28^.

The lack of a significant cardiovascular benefit from colchicine in our advanced CKD cohort, despite its proven efficacy in the general population, warrants explanation. This finding may be attributable to the high burden of noncardiovascular comorbidities in patients with advanced CKD, which can obscure the cardiovascular benefits of colchicine^29^. Furthermore, cardiovascular disease in patients with CKD is driven by distinct mechanisms that may be less responsive to colchicine’s anti-inflammatory effects—for example, vascular calcification, uremic toxin accumulation, and abnormal mineral metabolism^30,31^. The phenomenon of “reverse epidemiology,” whereby traditional cardiovascular risk factors and interventions behave differently in advanced CKD, may also explain the observations^32^. Progressive dysfunction in advanced CKD is driven by sustained inflammation, oxidative stress, and profibrotic pathways, with NLRP3 inflammasome activation playing a central role^23^. Experimental data suggest that colchicine inhibits this activation, thereby attenuating tubular injury and interstitial fibrosis^33^. This mechanism may make renal outcomes more responsive to anti-inflammatory intervention than cardiovascular endpoints. Notably, the observation that colchicine confers significant renoprotection despite limited cardiovascular benefit suggests that inflammation is a more critical driver of kidney disease progression than of cardiovascular outcomes in this population.

Although our findings indicate a renoprotective effect of colchicine, alternative explanations should be considered. Residual confounding is possible despite robust PSM because patients prescribed colchicine may have received relatively intensive care or close clinical monitoring. However, our subgroup and sensitivity analyses consistently revealed renoprotective benefits across models and patient subgroups, supporting the robustness of the observed association, although causality cannot be established. Another potential source of bias is that colchicine users may have had less severe AMI events than did nonusers; however, this bias was addressed by matching for troponin levels and excluding patients who underwent coronary artery bypass grafting within 6 months after the index date. Furthermore, if colchicine users had consistently experienced less severe AMI, a lower incidence of cardiovascular events would be expected in this group, an outcome that was not observed. The possibility of reverse causation also warrants attention, as declining renal function may have discouraged colchicine use rather than colchicine preserving kidney function. To address this selection bias, several safeguards were applied. PSM balanced baseline kidney function between groups (mean eGFR ~ 34–36 mL/min/1.73m^2^), and the absence of effects on MACE and all-cause mortality argues against the colchicine group being inherently healthier at baseline. Additionally, sensitivity analyses excluding prior colchicine users and landmark analyses both yielded consistent renoprotective benefits. Although residual confounding cannot be entirely excluded in an observational study, the consistency across models and subgroups supports the consistency of the observed association between early colchicine use and improved renal outcomes.

The present study unveils several key directions for future research. Our findings are compatible with the hypothesis that colchicine may mitigate inflammatory pathways relevant to kidney injury after AMI, but this study did not directly assess renal inflammation or fibrosis. Future investigations should explore the mechanisms through which colchicine confers renal protection—for example, its effects on renal cells and inflammatory pathways. Our results further indicate that anti-inflammatory therapies exert differential effects on cardiovascular and renal outcomes, highlighting the need for personalized strategies for treating advanced CKD. If validated, colchicine—given its safety profile and accessibility—could become a valuable therapeutic option for decelerating post-AMI CKD progression. The findings emphasize the need for trials focused on renal outcomes and mechanistic biomarker studies to better define colchicine’s role in patients with advanced CKD.

In contemporary cardiovascular practice, colchicine for secondary prevention after acute myocardial infarction is typically prescribed at a low dose of 0.5–0.6 mg once daily, as supported by large randomized trials such as COLCOT^14^ and LoDoCo2^15^. Recent cardiovascular guidelines have incorporated low-dose colchicine as a potential adjunct therapy for secondary prevention in selected patients with established atherosclerotic cardiovascular disease^34^. Although the TriNetX platform captures prescription records, it does not reliably provide detailed information on cumulative dose, exact daily dosing, or medication adherence across institutions. Therefore, while low-dose regimens are standard in post-AMI practice, precise dosing patterns and treatment duration could not be confirmed in this dataset and may have varied among patients.

Our study has several strengths. The matched cohort of 2,400 patients provided strong analytical power, though smaller subgroups limited significance in some populations. Consistent results across multiple methods and diverse TriNetX data enhance validity and generalizability. PSM minimized confounding, though residual bias cannot be excluded. Sensitivity and landmark analyses confirmed the robustness of our findings, and TriNetX EHRs ensured data completeness. However, this study has several limitations inherent to retrospective analyses of multi-institutional EHRs. First, documentation inconsistencies, missing data, and potential misclassification may occur; we mitigated these issues through stringent inclusion criteria and matching. Second, although TriNetX captures colchicine prescription records, the exact clinical indication for colchicine use, medication adherence, dose intensity, and treatment duration could not be reliably determined. Thus, colchicine may have been prescribed for post-AMI secondary prevention, gout, pericarditis, or other inflammatory conditions, raising the possibility of confounding by indication despite PSM and sensitivity analyses. Therefore, our findings should be interpreted as associations with colchicine prescription rather than confirmed indication-specific treatment effects. Third, the TriNetX platform did not uniformly provide detailed information on revascularization procedures or procedure-related contrast exposure. Therefore, although PSM was performed before outcome analysis, residual confounding related to AMI severity, revascularization, and contrast-associated renal injury cannot be fully excluded. Nonetheless, our objective was to evaluate colchicine effectiveness across the real-world spectrum of AMI severity, and thus the findings reflect outcomes in a broad post-AMI population. Fourth, the baseline etiology of CKD could not be definitively determined, as this information is not uniformly captured in the TriNetX database. However, we matched for the primary comorbidities associated with renal decline, including diabetes and hypertension, to ensure comparability between the cohorts. Fifth, colchicine prescription may be a proxy for higher health-care utilization and closer follow-up, which is not fully captured in TriNetX and may bias the observed association; therefore, our findings should be interpreted as associative rather than causal. Finally, the generalizability of these results is limited because about 50% of the participants are White. This majority representation may restrict the applicability of the findings to other racial or ethnic groups, making it difficult to extend the results to more diverse populations.

## Conclusion

In patients with advanced CKD, colchicine initiation within one month after AMI was associated with a lower risk of dialysis dependence over a maximum four-year follow-up. In landmark analyses, initiation within two weeks was associated with lower all-cause mortality. These findings support a possible renal benefit, whereas any survival benefit remains uncertain and requires prospective validation.

## Supplementary Information

Supplementary Information.

## Data Availability

TriNetX is a comprehensive network that connects a wide range of research centers, enabling collaboration and data sharing between institutions. This platform provides immediate access to large quantities of anonymized data, which are extracted from the electronic health records of numerous participating health-care organizations. By leveraging this resource, researchers can efficiently explore and analyze clinical information while maintaining strict patient confidentiality. All authorized users are able to conveniently access this rich dataset through the TriNetX online portal, which can be found at [https://live.trinetx.com, allowing for seamless integration into the research workflow. Access for this study was facilitated through the Shin-Kong Wu Ho-Su Memorial Hospital, a participating institution connected to the TriNetX network.
