# Supplementary material for: Colchicine use after acute myocardial infarction is associated with reduced dialysis dependence in advanced chronic kidney disease
Source: Sci Rep. 2026 Jun 4;16:25626. doi: 10.1038/s41598-026-54682-y (PMC13478514; doi:10.1038/s41598-026-54682-y)
Supplement: Supplementary file 1 [file 41598_2026_54682_MOESM1_ESM.docx]

**Supplementary Online Content**

[**eMethod.** Codes for cohorts, outcomes, and baseline data	2](#_Toc222176177)

[**1.Definition for cohorts**	2](#_Toc222176178)

[**2.Codes for outcome definitions**	7](#_Toc222176179)

[**3.Codes for the baseline covariates for propensity score matching**	8](#_Toc222176180)

[**eMethod 2** The detail of propensity score matching in TriNetX platform	11](#_Toc222176181)

[**Figure S1.** Schematic of study design	18](#_Toc222176182)

[**Figure S2**. Time distribution of studied cohorts	19](#_Toc222176183)

[**Table S1.** Adverse effect analysis of medications	20](#_Toc222176184)

[**Table S2**. Clinical outcomes in different propensity score matching models.	21](#_Toc222176185)

[**Table S3**. Clinical outcomes across various follow-up periods	23](#_Toc222176186)

[**Table S4**. Sensitivity test by excluding the colchicine use before index date	25](#_Toc222176187)

[**Table S5**. Sensitivity test by including more baseline parameters	27](#_Toc222176188)

# **eMethod.** Codes for cohorts, outcomes, and baseline data

## **1.Definition for cohorts**

**Query Criteria for Cohort 1 (Colchicine user within one month after AMI in advanced CKD)**

| Group 1 | | | | | |
| --- | --- | --- | --- | --- | --- |
|  | **visit>=3** | | | | |
|  | must have |  | visit | TNX: Visit | Visit |
|  | number of instances | | Greater than or equal to 3 instances | | |
|  | date constraint | | The terms in this group occurred at any time | | |
| Group 2 | | | | | |
|  | **Advanced CKD** | | | | |
|  | must have |  | laboratory | TNX:8001 | Glomerular filtration rate/1.73 sq M.predicted [Volume Rate/Area] in Serum, Plasma or Blood by Creatinine-based formula (MDRD) (at least 18 years old at event; at most 45.00 mL/min/{1.73_m2} (most recent occurrence)) |
|  | date constraint | | The terms in this group occurred between Jan 1, 2012 and Dec 31, 2022 | | |
| Group 3 | | | | | |
|  | **Group 3A advanced CKD** | | | | |
|  | must have |  | laboratory | TNX:8001 | Glomerular filtration rate/1.73 sq M.predicted [Volume Rate/Area] in Serum, Plasma or Blood by Creatinine-based formula (MDRD) (at least 18 years old at event; at most 45.00 mL/min/{1.73_m2}) |
|  | date constraint | | The terms in this group occurred between Jan 1, 2012 and Dec 31, 2022 | | |
|  | event relationship | | Any instance of Group 3B occurred on or after the first instance of advanced CKD | | |
|  | **Group 3B. Those with AMI** | | | | |
|  | must have |  | diagnosis | UMLS:ICD10CM:I21 | Acute myocardial infarction |
| Group 4 | | | | | |
|  | **Group 4A advanced CKD** | | | | |
|  | must have |  | diagnosis | UMLS:ICD10CM:I21 | Acute myocardial infarction |
|  | date constraint | | The terms in this group occurred between Jan 1, 2012 and Dec 31, 2022 | | |
|  | event relationship | | Any instance of Group 4B occurred before or up to 1 month after the first instance of advanced CKD | | |
|  | **Group 4B exclude neoplasms** | | | | |
|  | cannot have |  | diagnosis | UMLS:ICD10CM:C00-D49 | Neoplasms |
| Group 5 | | | | | |
|  | **Group 5A advanced CKD** | | | | |
|  | must have |  | diagnosis | UMLS:ICD10CM:I21 | Acute myocardial infarction |
|  | date constraint | | The terms in this group occurred between Jan 1, 2012 and Dec 31, 2022 | | |
|  | event relationship | | Any instance of Group 5B occurred before or up to 1 month after the first instance of advanced CKD | | |
|  | **Group 5B** | | | | |
|  | cannot have |  | diagnosis | UMLS:ICD10CM:Z94 | Transplanted organ and tissue status |
| Group 6 | | | | | |
|  | **Group 6A advanced CKD** | | | | |
|  | must have |  | diagnosis | UMLS:ICD10CM:I21 | Acute myocardial infarction |
|  | date constraint | | The terms in this group occurred between Jan 1, 2012 and Dec 31, 2022 | | |
|  | event relationship | | Any instance of Group 6B occurred within 5 months before or up to 1 month after the first instance of advanced CKD | | |
|  | **Group 6B exclude dialysis** | | | | |
|  | cannot have |  | procedure | UMLS:CPT:1012740 | Dialysis Services and Procedures |
|  |  | or | procedure | UMLS:CPT:90945 | Dialysis procedure other than hemodialysis (eg, peritoneal dialysis, hemofiltration, or other continuous renal replacement therapies), with single evaluation by a physician or other qualified health care professional |
| Group 7 | | | | | |
|  | **Group 7A advanced CKD** | | | | |
|  | must have |  | diagnosis | UMLS:ICD10CM:I21 | Acute myocardial infarction |
|  | date constraint | | The terms in this group occurred between Jan 1, 2012 and Dec 31, 2022 | | |
|  | event relationship | | Any instance of Group 7B occurred within 5 months before or up to 1 month after the first instance of advanced CKD | | |
|  | **Group 7B exclude CABG** | | | | |
|  | cannot have |  | diagnosis | UMLS:ICD10CM:Z95.1 | Presence of aortocoronary bypass graft |
| Group 8 | | | | | |
|  | **Group 8A advanced CKD** | | | | |
|  | must have |  | diagnosis | UMLS:ICD10CM:I21 | Acute myocardial infarction |
|  | date constraint | | The terms in this group occurred between Jan 1, 2012 and Dec 31, 2022 | | |
|  | event relationship | | Any instance of Group 8B occurred within 1 month on or after the first instance of advanced CKD | | |
|  | **Group 8B Colchicine user** | | | | |
|  | must have |  | medication | NLM:RXNORM:2683 | colchicine |

**Query Criteria for Cohort 1 (Colchicine non-user within one month after AMI in advanced CKD)**

| Group 1 | | | | | |
| --- | --- | --- | --- | --- | --- |
|  | **visit>=3** | | | | |
|  | must have |  | visit | TNX:Visit | Visit |
|  | number of instances | | Greater than or equal to 3 instances | | |
|  | date constraint | | The terms in this group occurred at any time | | |
| Group 2 | | | | | |
|  | **advanced CKD** | | | | |
|  | must have |  | laboratory | TNX:8001 | Glomerular filtration rate/1.73 sq M.predicted [Volume Rate/Area] in Serum, Plasma or Blood by Creatinine-based formula (MDRD) (at least 18 years old at event; at most 45.00 mL/min/{1.73_m2} (most recent occurrence)) |
|  | date constraint | | The terms in this group occurred between Jan 1, 2012 and Dec 31, 2022 | | |
| Group 3 | | | | | |
|  | **Group 3A advanced CKD** | | | | |
|  | must have |  | laboratory | TNX:8001 | Glomerular filtration rate/1.73 sq M.predicted [Volume Rate/Area] in Serum, Plasma or Blood by Creatinine-based formula (MDRD) (at least 18 years old at event; at most 45.00 mL/min/{1.73_m2}) |
|  | date constraint | | The terms in this group occurred between Jan 1, 2012 and Dec 31, 2022 | | |
|  | event relationship | | Any instance of Group 3B occurred on or after the first instance of advanced CKD | | |
|  | **Group 3B Those with AMI** | | | | |
|  | must have |  | diagnosis | UMLS:ICD10CM:I21 | Acute myocardial infarction |
| Group 4 | | | | | |
|  | **Group 4A advanced CKD** | | | | |
|  | must have |  | diagnosis | UMLS:ICD10CM:I21 | Acute myocardial infarction |
|  | date constraint | | The terms in this group occurred between Jan 1, 2012 and Dec 31, 2022 | | |
|  | event relationship | | Any instance of Group 4B occurred before or up to 1 month after the first instance of advanced CKD | | |
|  | **Group 4B exclude neoplasm** | | | | |
|  | cannot have |  | diagnosis | UMLS:ICD10CM:C00-D49 | Neoplasms |
| Group 5 | | | | | |
|  | **Group 5A advanced CKD** | | | | |
|  | must have |  | diagnosis | UMLS:ICD10CM:I21 | Acute myocardial infarction |
|  | date constraint | | The terms in this group occurred between Jan 1, 2012 and Dec 31, 2022 | | |
|  | event relationship | | Any instance of Group 5B occurred before or up to 1 month after the first instance of advanced CKD | | |
|  | **Group 5B** | | | | |
|  | cannot have |  | diagnosis | UMLS:ICD10CM:Z94 | Transplanted organ and tissue status |
| Group 6 | | | | | |
|  | **Group 6A advanced CKD** | | | | |
|  | must have |  | diagnosis | UMLS:ICD10CM:I21 | Acute myocardial infarction |
|  | date constraint | | The terms in this group occurred between Jan 1, 2012 and Dec 31, 2022 | | |
|  | event relationship | | Any instance of Group 6B occurred within 5 months before or up to 1 month after the first instance of advanced CKD | | |
|  | **Group 6B exclude dialysis** | | | | |
|  | cannot have |  | procedure | UMLS:CPT:1012740 | Dialysis Services and Procedures |
|  |  | or | procedure | UMLS:CPT:90945 | Dialysis procedure other than hemodialysis (eg, peritoneal dialysis, hemofiltration, or other continuous renal replacement therapies), with single evaluation by a physician or other qualified health care professional |
| Group 7 | | | | | |
|  | **Group 7A advanced CKD** | | | | |
|  | must have |  | diagnosis | UMLS:ICD10CM:I21 | Acute myocardial infarction |
|  | date constraint | | The terms in this group occurred between Jan 1, 2012 and Dec 31, 2022 | | |
|  | event relationship | | Any instance of Group 7B occurred within 5 months before or up to 1 month after the first instance of advanced CKD | | |
|  | **Group 7B exclude CABG** | | | | |
|  | cannot have |  | diagnosis | UMLS:ICD10CM:Z95.1 | Presence of aortocoronary bypass graft |
| Group 8 | | | | | |
|  | **Group 8A advanced CKD** | | | | |
|  | must have |  | diagnosis | UMLS:ICD10CM:I21 | Acute myocardial infarction |
|  | date constraint | | The terms in this group occurred between Jan 1, 2012 and Dec 31, 2022 | | |
|  | event relationship | | Any instance of Group 8B occurred within 1 month on or after the first instance of advanced CKD | | |
|  | **Group 8B Colchicine non-users** | | | | |
|  | cannot have |  | medication | NLM:RXNORM:2683 | colchicine |

## **2.Codes for outcome definitions**

| Table below outlines the definitions for each outcome and the analysis specifications. For outcome definitions consisting of more than one term, at least one term must match. | | | |
| --- | --- | --- | --- |
| Dialysis dependence | | | |
|  | Diagnosis | UMLS:ICD10CM:Z99.2 | Dependence on renal dialysis |
| Mortality | | | |
|  | Demographics | Deceased | Deceased |
| Stroke | | | |
|  | Diagnosis | UMLS:ICD10CM:I63 | Cerebral infarction |
|  | Diagnosis | UMLS:ICD10CM:I61 | Nontraumatic intracerebral hemorrhage |
|  | Diagnosis | UMLS:ICD10CM:I62 | Other and unspecified nontraumatic intracranial hemorrhage |
| AMI | | | |
|  | Diagnosis | UMLS:ICD10CM:I21 | Acute myocardial infarction |
| Cardiogenic shock | | | |
|  | Diagnosis | UMLS:ICD10CM:R57.0 | Cardiogenic shock |
| Af and AF | | | |
|  | Diagnosis | UMLS:ICD10CM:I48 | Atrial fibrillation and flutter |
| HF | | | |
|  | Diagnosis | UMLS:ICD10CM:I50 | Heart failure |
| Cardiac arrest | | | |
|  | Diagnosis | UMLS:ICD10CM:I46 | Cardiac arrest |
| MACE | | | |
|  | Diagnosis | UMLS:ICD10CM:I21 | Acute myocardial infarction |
|  | Diagnosis | UMLS:ICD10CM:I63 | Cerebral infarction |
|  | Diagnosis | UMLS:ICD10CM:I61 | Nontraumatic intracerebral hemorrhage |
|  | Diagnosis | UMLS:ICD10CM:I62 | Other and unspecified nontraumatic intracranial hemorrhage |
|  | Diagnosis | UMLS:ICD10CM:I50 | Heart failure |
|  | Diagnosis | UMLS:ICD10CM:I46 | Cardiac arrest |
|  | Demographics | Deceased | Deceased |

## **3.Codes for the baseline covariates for propensity score matching**

| **Demographics** | | | | |  |  |  |  |  |  |
| --- | --- | --- | --- | --- | --- | --- | --- | --- | --- | --- |
|  | AI | Age at Index | | |  |  |  |  |  |  |
|  | 2106-3 | White people | | |  |  |  |  |  |  |
|  | 2054-5 | African American | | |  |  |  |  |  |  |
|  | 2028-9 | Asian | | |  |  |  |  |  |  |
|  | 2131-1 | Other Race | | |  |  |  |  |  |  |
|  | M | Male | | |  |  |  |  |  |  |
| **Diagnosis** | | | | |  |  |  |  |  |  |
| International Classification of Diseases, Tenth Revision, | | Name | | |  |  |  |  |  |  |
|  | E78 | Disorders of lipoprotein metabolism and other lipidemias | | |  |  |  |  |  |  |
|  | I10-I1A | Hypertensive diseases | | |  |  |  |  |  |  |
|  | I50 | Heart failure | | |  |  |  |  |  |  |
|  | I48 | Atrial fibrillation and flutter | | |  |  |  |  |  |  |
|  | I42 | Cardiomyopathy | | |  |  |  |  |  |  |
|  | I60-I69 | Cerebrovascular diseases | | |  |  |  |  |  |  |
|  | I26-I28 | Pulmonary heart disease and diseases of pulmonary circulation | | |  |  |  |  |  |  |
|  | I05-I09 | Chronic rheumatic heart diseases | | |  |  |  |  |  |  |
|  | E00-E07 | Disorders of thyroid gland | | |  |  |  |  |  |  |
|  | K74 | Fibrosis and cirrhosis of liver | | |  |  |  |  |  |  |
|  | J40-J4A | Chronic lower respiratory diseases | | |  |  |  |  |  |  |
|  | Z72.0 | Tobacco use | | |  |  |  |  |  |  |
|  | F17 | Nicotine dependence | | |  |  |  |  |  |  |
|  | F10 | Alcohol related disorders | | |  |  |  |  |  |  |
|  | E08-E13 | Diabetes mellitus | | |  |  |  |  |  |  |
|  | I49 | Other cardiac arrhythmias | | |  |  |  |  |  |  |
|  | K76.0 | Fatty (change of) liver, not elsewhere classified | | |  |  |  |  |  |  |
| **Medication** | | | | |  |  |  |  |  |  |
| Anatomical Therapeutic Chemical | | Name | | |  |  |  |  |  |  |
|  | A10A | INSULINS AND ANALOGUES | | |  |  |  |  |  |  |
|  | C10AA | HMG CoA reductase inhibitors | | |  |  |  |  |  |  |
|  | C10AB | Fibrates | | |  |  |  |  |  |  |
|  | C09 | AGENTS ACTING ON THE RENIN-ANGIOTENSIN SYSTEM | | |  |  |  |  |  |  |
|  | C03 | DIURETICS | | |  |  |  |  |  |  |
|  | C07 | BETA BLOCKING AGENTS | | |  |  |  |  |  |  |
|  | C08 | CALCIUM CHANNEL BLOCKERS | | |  |  |  |  |  |  |
|  | C04 | PERIPHERAL VASODILATORS | | |  |  |  |  |  |  |
|  | B01 | ANTITHROMBOTIC AGENTS | | |  |  |  |  |  |  |
|  | A10B | BLOOD GLUCOSE LOWERING DRUGS, EXCL. INSULINS | | |  |  |  |  |  |  |
| **Laboratory data** | | | | |  |  | | N05 | | PSYCHOLEPTICS |
| TNX, Curated | | Name (unit) | Missing rate after PSM matching | |  | | ANALGESICS | |  |  |
|  |  |  | Colchicine users | Colchicine nonusers |  |  |  |  |  |  |
|  | 9029 | Sodium in Serum (mmol/L) | 3.2% | 5.5% |  |  |  |  |  |  |
|  | 9022 | Calcium in Serum (mg/dL) | 6% | 8.7% |  |  |  |  |  |  |
|  | 9027 | Phosphate in Serum (mg/dL) | 54.9% | 56.8% |  |  |  |  |  |  |
|  | 9014 | Hemoglobin in Blood (g/dL) | 6.1% | 8.4% |  |  |  |  |  |  |
|  | 9044 | Alanine aminotransferase in Serum | 21.9% | 24.3% |  |  |  |  |  |  |
|  | 9047 | Aspartate aminotransferase in Serum (U/L) | 22.5% | 24.5% |  |  |  |  |  |  |
|  | 9045 | Albumin in Serum (g/dL) | 21.4% | 24.8% |  |  |  |  |  |  |
|  | 9002 | Cholesterol in LDL in Serum (mg/dL) | 63% | 66% |  |  |  |  |  |  |
|  | 9001 | Cholesterol in HDL in Serum (mg/dL) | 62.7% | 65.8% |  |  |  |  |  |  |
|  | 9004 | Triglyceride in Serum (mg/dL) | 62.7% | 65.3% |  |  |  |  |  |  |
|  | 9037 | Hemoglobin A1c/Hemoglobin.total in Blood (%) | 56% | 57.8% |  |  |  |  |  |  |
|  | 9083 | BMI (kg/m^2^) | 30.8% | 32.5% |  |  |  |  |  |  |
|  | LG34791-0 | Protein/Creatinine in Urine | 95.5% | 96.8% |  |  |  |  |  |  |
|  | 9005 | Troponin I.cardiac in Serum, Plasma or Blood | 43.5% | 53% |  |  |  |  |  |  |
|  | 9028 | Potassium in Serum (mmol/L) | 5.8% | 7.2% |  |  |  |  |  |  |
|  | 8001 | Glomerular filtration rate/1.73 sq M.predicted in Serum, by Creatinine-based formula (MDRD) (mL·min⁻¹·1.73 m⁻²) | 1.8% | 3.9% |  |  |  |  |  |  |

# **eMethod 2** The detail of propensity score matching in TriNetX platform

**(https://support.trinetx.com/hc/en-us/articles/360011978033)**

To conduct PSM using the TNX Research platform, you must first identify two cohorts of interest, index events, outcomes of interest, and attributes of patients which may act as confounders to the outcomes of interest. For the purpose of this article, these attributes will be called “covariates.”

Within Balance, when you run a propensity score matching analysis, the system conducts a propensity score matching to balance the cohorts:

1. For each patient in each cohort, the system computes values for each covariate.
2. These data form a matrix of covariate values for each
3. The system performs a logistic regression on the pooled matrices, to “predict” which cohort each patient originates from. The value of this model for a patient is that patient’s predicted probability of being in the second cohort or “propensity score.”
4. For each patient in the smaller cohort, the system chooses as match from the larger cohort (if any patients in the larger cohort are close enough). The pairs then form a subset of each cohort.

Within Outcomes, when you run compare cohorts after matching, the system conducts a propensity score matching to compare outcomes between the balance the cohorts:

1. For each patient in each cohort, the system computes both the outcome(s) of interest and values for each covariate.
2. These data form a matrix of covariate values for each
3. The system performs a logistic regression on the pooled matrices, to “predict” which cohort each patient originates from. The value of this model for a patient is that patient’s predicted probability of being in the second cohort or “propensity score.”
4. For each patient in the smaller cohort, the system chooses as match from the larger cohort (if any patients in the larger cohort are close enough). The pairs then form a subset of each cohort.
5. The system compares outcomes on these after matching subsets, rather than the original cohorts.

**Values in the Covariate Matrix**

In the covariate matrix, each row represents one patient, and each column represents one covariate. Each cell contains exactly one non-null numerical value.

 All covariates are one of the following forms:

- **Binary:** yes/no;
- **Categorical:** real values placed in categories based on their value; or
- **Continuous:** real values represented directly.

When a covariate is binary (for example, a patient having history of asthma), the cell for that patient and covariate is set to 0 for “not present” or 1 for “is present.”

When a covariate is categorical, the categories are specified as ranges that the continuous variable can take (for example, having a value for a Sodium lab between 130 and 140). Each category becomes a distinct covariate which is either 0 for “not present” or 1 for “is present.” It is possible for all columns to be 0 (for example if there are no values in the time window, or if all values are outside all the categories). It is also possible for multiple values to be 1 (for example if there are multiple values in the time window, or if the categories overlap).

When a covariate is continuous (for example, current age, or age at time of the index event), then the value for that patient (if any) is placed in the relevant cell.

**Details on Scoring and Logistic Regression**

When you run propensity score matching, the system generates a propensity score for each patient in each cohort. The propensity score ranges between 0 and 1 and indicates the predicted probability a patient is in cohort B given the patient’s covariates.

To uses logistic regression to generate the propensity scores, through an implementation of the well-tested, standard software package [scikit-learn](https://scikit-learn.org/). The code used in production is as follows (some logging, etc. removed for clarity):

import numpy as np

from sklearn.linear_model import LogisticRegression

from sklearn.preprocessing import Imputer

from impute import fill_nans

def fill_nans(data, axis=0):

  imp = Imputer(missing_values='NaN', strategy=method, axis=axis)

    return imp.fit_transform(data)

def propensity_scores(matrix_a, matrix_b):

matrix_a = fill_nans(matrix_a, method='mean')

matrix_b = fill_nans(matrix_b, method='mean')

full_matrix = np.concatenate((matrix_a, matrix_b), axis=0)

\# cohort A is "zero" and cohort B is "one" from the regression’s perspective

target_a = np.zeros(matrix_a.shape[0], dtype=float)

target_b = np.ones(matrix_b.shape[0], dtype=float)

target = np.concatenate([target_a, target_b])

lr = LogisticRegression(C=1000)

lr.fit(full_matrix, target)

def get_scores(X):

\# .predict_proba returns [prob_class_zero, prob_class_one] for each row,

\# while the score we want is the probability of being in class B

full_scores = lr.predict_proba(X)

return full_scores[:, 1]

return (get_scores(matrix_a), get_scores(matrix_b))

Here the “score” for each patient is the regression model applied to that patient’s row. Informally, this is the probability that the patient belongs to cohort B, based on the cohorts it has seen.

Here “matrix_a” is the covariate matrix for the cohort you selected as Cohort A. Likewise, “matrix_b” is the covariate matrix for the cohort you selected as Cohort B.  Data are pooled across all HCOs, so all patients in the analysis are a row in one of these matrices. Note that the code instructs the system to replace all missing (NaN) values in each matrix with the mean from that column; however, at time of writing all covariates are either binary, categorical (which expands to a set of binary columns), or continuous but guaranteed to exist, so this imputation is vacuous.

The call “LogisticRegression(C=1000)” means we predict the probability of being in cohort B using logistic regression. The value of C means we weight the mean residual by a factor of 1000 as compared to the norm of the coefficients. This means we are using a very small amount of L2 (ridge) regression, which is needed to make the objective function convex (so the regression converges), but with such a small value, it has very little effect on the model. Due to the very small amount of regularization and the scale of our covariates being constrained, it is not necessary to perform mean normalization as a preprocessing step.

**Matching Details**

Once the system has generated a propensity score for each patient, the system performs matching to identify the matched subsets. We use “greedy nearest neighbor matching” with a caliper of 0.1 pooled standard deviations. Sample code is here:

import numpy as np

def _pooled_sd(floats_a, floats_b):

var_a = np.var(floats_a)

var_b = np.var(floats_b)

return sqrt((var_a + var_b) / 2.0)

def _naive_nearest_neighbor_match(scores_a, scores_b, caliper):

\# Perform nearest neighbor matching. Assume that len(scores_a) <=len(scores_b)

num_a, num_b = len(scores_a), len(scores_b)

max_diff = _pooled_sd(scores_a, scores_b) * caliper

chosen_a = np.zeros(num_a, dtype=bool) # True if the patient is matched, start false

chosen_b = np.zeros(num_b, dtype=bool)

for a_ind in range(0, num_a):

best_diff = max_diff

best_b_ind = None

found_any = False

for b_ind in range(0, num_b):

if chosen_b[b_ind]:

continue

diff = abs(scores_a[a_ind] - scores_b[b_ind])

if diff < best_diff or (diff == best_diff and not found_any):

best_diff = diff

best_b_ind = b_ind

found_any = True

if best_b_ind is not None:

chosen_a[a_ind] = True

chosen_b[best_b_ind] = True

return (chosen_a, chosen_b)

*Note:* the code used in production is highly optimized and significantly longer. The above code is taken directly from our test fixtures and is used to ensure that the optimized code produces identical results to the above simplified code.

We use a “caliper” of 0.1 pooled standard deviations of the propensity scores in aggregate, which means that patients with very different propensity scores are not matched. The system matches patients using the following algorithm:

1. For each patient in cohort A (assumed to be the smaller of the two), and identify the patient whose score is closest to the patient in cohort A from the patients in cohort B who have not yet been matched.
2. If a match is found, mark both as “chosen” and move on.
3. At the end, return the labels of each cohort, indicating which patients were chosen and which were not. The patients who were chosen form the “matched” cohorts.

**Pooling the Cohorts, and Compensating for Unbalanced Cohorts across HCOs**

TriNetX pulls data from a federated data network made up of many healthcare organizations across the world. Each site computes a covariate matrix for the patients they contribute to the analysis and send it to a central processing point to be pooled and analyzed as a single matrix.

The order of the rows in the matrix should not impact the propensity scores generated for each patient; logistic regression is highly stable with respect to permuting the rows.  In contrast, nearest neighbor matching, can be influenced by the order of rows in the matrix. For example, if two identical patients in cohort B match equally well to a patient in cohort A, the first will be chosen and the second will not.  Therefore, if the order of the rows carries some information (perhaps as an artifact of the pooling), the order can introduce bias.

To eliminate this bias, we randomize the order of the records in the covariate matrix.  We sort the provider results by the unique ID of the HCO (so the information of “which HCO responded first” is deleted), concatenate the matrices into one large matrix, then shuffle them using np.random.shuffle(full_matrix_a) and np.random.shuffle(full_matrix_b). To assure determinism, a call to np.random.seed(FIXED_SEED) precedes all calls to shuffle, so that successive runs do not change unless the underlying data changes.

# **Figure S1.** Schematic of study design

# **Figure S2**. Time distribution of studied cohorts

| **Table S1.** Adverse effect analysis of medications | | | | | | |
| --- | --- | --- | --- | --- | --- | --- |
| Adverse effects | After propensity score matching; with a maximum follow-up of 4-year | | | | | |
|  | Colchicine user  within one month  (n = 1,035) | | Colchicine nonusers  within one month  (n = 1,035) | | Colchicine users vs. nonusers | |
|  | Events (n) | (%) | Events (n) | (%) | HR (95%CI) | *p* value |
| Myopathy | 24 | 2.3 | 20 | 1.9 | 1.08 (0.59–1.95) | 0.808 |
| Bone marrow suppression | 119 | 11.5 | 95 | 9.2 | 1.14 (0.871.49) | 0.341 |
| Hepatotoxicity | 20 | 1.9 | 17 | 1.6 | 1.06 (0.55–2.02) | 0.863 |
| Diarrhea | 123 | 11.9 | 110 | 10.6 | 1.02 (0.79–1.32) | 0.874 |
| Abbreviation: HR, hazard ratio; CI, confidence interval. | | | | | | |

| **Table S2**. Clinical outcomes in different propensity score matching models. | | | | | | |
| --- | --- | --- | --- | --- | --- | --- |
| Clinical Outcomes | Colchicine users versus nonusers within one month after AMI; with a maximum follow-up of 4-year | | | | | |
|  | Model 1 | | Model 2 | | Model 3 | |
|  | HR (95%CI) | *P value* | HR (95%CI) | *P value* | HR (95%CI) | *P value* |
| **Primary outcome** |  |  |  |  |  |  |
| Dialysis dependence | 0.88 (0.7–1.11) | 0.292^†^ | 0.83 (0.66–1.04) | 0.105^†^ | 0.79 (0.63–0.98) | 0.035 |
| **Secondary outcomes** |  |  |  |  |  |  |
| MACE^#^ | 1.17 (1.05–1.30) | 0.004 | 1.05 (0.95–1.16) | 0.312 | 1.04 ( 0.94–1.14) | 0.492 |
| Mortality | 1.17 (0.97–1.41) | 0.097 | 1.04 (0.88–1.23) | 0.649 | 0.96 ( 0.82–1.14) | 0.658 |
| **Specified analysis** |  |  |  |  |  |  |
| Stroke | 0.8 (0.62–1.03 ) | 0.079 | 0.9 (0.70–1.16) | 0.425 | 0.7 (0.55–0.90) | 0.005 |
| AMI | 1.13 (0.97–1.32) | 0.116 | 0.97 (0.84–1.12) | 0.675 | 1.00 (0.86–1.15) | 0.948 |
| Af /AF | 1.42 (1.21–1.68) | <0.001 | 1.15 (0.99–1.33) | 0.074 | 1.1 (0.95–1.27) | 0.214 |
| Heart failure | 1.22 (1.08–1.38) | 0.001 | 1.06 (0.95–1.19) | 0.292 | 1.1 (0.98–1.24) | 0.093 |
| Cardiac arrest | 1.33 (0.91–1.97) | 0.144 | 1.01 (0.72–1.43) | 0.951 | 1.02 (0.72–1.44) | 0.919 |
| Model 1 uses age, sex, race, and BMI for propensity score matching. Model 2 adds baseline comorbidities to model 1, while model 3 further includes baseline medication use.  ^#^MACE included acute myocardial infarction, stroke, heart failure and death.  ^†^This indicate the proportional hazard assumption is violated.  Abbreviation: HR, hazard ratio; CI, confidence interval; MACE, major adverse cardiovascular events; AMI, acute myocardial infarction; Af/AF, atrial fibrillation and atrial flutter. | | | | | | |

| **Table S3**. Clinical outcomes across various follow-up periods | | | | | | |
| --- | --- | --- | --- | --- | --- | --- |
| Clinical Outcomes | Colchicine users versus nonusers within one month after AMI; propensity score matching^&^ | | | | | |
|  | 1 month to 1 years | | 1 month to 2 years | | 1 month to 3 years | |
|  | HR (95%CI) | *P value* | HR (95%CI) | *P value* | HR (95%CI) | *P value* |
| **Primary outcome** |  |  |  |  |  |  |
| Dialysis dependence | 0.73 (0.56–0.95) | 0.018 | 0.81 (0.62–1.04) | 0.102 | 0.74 (0.59–0.93) | 0.009 |
| **Secondary outcome** |  |  |  |  |  |  |
| MACE^#^ | 1.06 ( 0.95–1.19 ) | 0.312^†^ | 1.04 ( 0.93–1.16 ) | 0.505^†^ | 0.98 ( 0.88–1.09 ) | 0.687 |
| Mortality | 1.04 ( 0.79–1.38 ) | 0.771 | 0.99 ( 0.8–1.24 ) | 0.937 | 0.9 ( 0.74–1.08 ) | 0.248 |
| **Specified analysis** |  |  |  |  |  |  |
| Stroke | 0.85 ( 0.59–1.23 ) | 0.394 | 0.67 ( 0.5–0.91 ) | 0.009 | 0.78 ( 0.58–1.04 ) | 0.085 |
| AMI | 1 ( 0.84–1.2 ) | 0.979 | 1.09 ( 0.92–1.28 ) | 0.334 | 0.97 ( 0.84–1.13 ) | 0.716 |
| Af and AF | 1.15 ( 0.96–1.37 ) | 0.124^†^ | 1.04 ( 0.88–1.24 ) | 0.615 | 1.07 ( 0.91–1.25 ) | 0.399^†^ |
| Heart failure | 1.08 ( 0.95–1.23 ) | 0.226 | 1.1 ( 0.97–1.25 ) | 0.130^†^ | 1.05 ( 0.93–1.18 ) | 0.467 |
| Cardiac arrest | 1.33 ( 0.78–2.26 ) | 0.293 | 0.84 ( 0.55–1.29 ) | 0.429 | 1.19 ( 0.8–1.75 ) | 0.393 |
| ^&^The propensity score matching includes all parameters in table 1 of main article to achieve balanced cohorts.  ^#^MACE included acute myocardial infarction, stroke, heart failure and death.  ^†^This indicate the proportional hazard assumption is violated.  Abbreviation: HR, hazard ratio; CI, confidence interval; MACE, major adverse cardiovascular events; AMI, acute myocardial infarction; Af/AF, atrial fibrillation and atrial flutter. | | | | | | |

| **Table S4**. Sensitivity test by excluding the colchicine use before index date | | | | | | |
| --- | --- | --- | --- | --- | --- | --- |
| Clinical Outcomes | After propensity score matching^&^; with a maximum follow-up of 4-year | | | | | |
|  | Colchicine users  within one month  (n = 828) | | Colchicine nonusers  within one month  (n = 828) | | Colchicine users vs. Nonusers | |
|  | Events | % | Events | % | HR (95%CI) | *P value* |
| **Primary outcome** |  |  |  |  |  |  |
| Dialysis dependence | 126 | 15.2 | 151 | 18.2 | 0.74 (0.58–0.94) | **0.012** |
| **Secondary outcome** |  |  |  |  |  |  |
| MACE^#^ | 585 | 70.7 | 542 | 65.5 | 1.03 (0.92–1.16) | 0.636 |
| Mortality | 235 | 28.4 | 210 | 25.4 | 1.01 (0.84–1.22) | 0.905 |
| **Specified analysis** |  |  |  |  |  |  |
| Stroke | 75 | 9.1 | 89 | 10.7 | 0.75 (0.55–1.02) | 0.065 |
| AMI | 280 | 55.8 | 268 | 52.6 | 0.96 (0.81–1.14) | 0.629 |
| Af and AF | 277 | 33.5 | 264 | 31.9 | 0.97 (0.82–1/15) | 0.737 |
| Heart failure | 457 | 55.2 | 422 | 51.0 | 1.03 (0.89–1.17) | 0.701 |
| Cardiac arrests | 53 | 6.4 | 54 | 6.5 | 0.89 (0.61–1.29) | 0.528 |
| ^&^The propensity score matching includes all parameters in table 1 of main article to achieve balanced cohorts.  ^#^MACE included myocardial infarction, stroke, heart failure, and death  Abbreviation: HR, hazard ratio ; CI, confidence interval; MACE, major adverse cardiovascular events; AMI, acute myocardial infarction; Af, atrial fibrillation; AF, atrial flutter. | | | | | | |

| **Table S5**. Sensitivity test by including more baseline parameters | | | | | | |
| --- | --- | --- | --- | --- | --- | --- |
| Clinical Outcomes | After propensity score matching^&^; with a maximum follow-up of 4-year | | | | | |
|  | Colchicine users  within one month  (n = 986) | | Colchicine nonusers  within one month  (n = 986) | | Colchicine users vs. Nonusers | |
|  | Events | % | Events | % | HR (95%CI) | *P value* |
| **Primary outcome** |  |  |  |  |  |  |
| Dialysis dependence | 143 | 14.5 | 164 | 16.6 | 0.80 (0.64–0.99) | **0.044** |
| **Secondary outcome** |  |  |  |  |  |  |
| MACE^#^ | 707 | 71.7 | 661 | 67.0 | 1.03 (0.92–1.14) | 0.620 |
| Mortality | 279 | 28.3 | 257 | 26.1 | 1.01 (0.85–1.19) | 0.930 |
| **Specified analysis** |  |  |  |  |  |  |
| Stroke | 100 | 10.1 | 83 | 8.4 | 1.12 (0.84–1.50) | 0.431 |
| AMI | 342 | 34.7 | 349 | 35.4 | 0.89 (0.76–1.03) | 0.115 |
| Af and AF | 345 | 34.9 | 307 | 31.1 | 1.09 (0.94–1.27) | 0.267 |
| Heart failure | 558 | 56.6 | 527 | 54.4 | 1.03 (0.92–1.16) | 0.624 |
| Cardiac arrests | 65 | 6.6 | 55 | 5.6 | 1.1 (0.77–1.58) | 0.603 |
| ^&^ Propensity score matching used the same parameters as in Table 1 of the main article, with additional matching variables including gout history, nonsteroidal anti-inflammatory drug use, serum urate, C-reactive protein, and erythrocyte sedimentation rate.  ^#^MACE included myocardial infarction, stroke, heart failure, and death  Abbreviation: HR, hazard ratio ; CI, confidence interval; MACE, major adverse cardiovascular events; AMI, acute myocardial infarction; Af, atrial fibrillation; AF, atrial flutter. | | | | | | |
